# Antibody cocktail effective against variants of SARS-CoV-2

**DOI:** 10.1186/s12929-021-00777-9

**Published:** 2021-11-23

**Authors:** Kang-Hao Liang, Pao-Yin Chiang, Shih-Han Ko, Yu-Chi Chou, Ruei-Min Lu, Hsiu-Ting Lin, Wan-Yu Chen, Yi-Ling Lin, Mi-Hua Tao, Jia-Tsrong Jan, Han-Chung Wu

**Affiliations:** 1 Biomedical Translation Research Center (BioTReC), Academia Sinica, Taipei, 11529 Taiwan; 2 Institute of Cellular and Organismic Biology, Academia Sinica, No. 128, Academia Road, Section 2, Nankang, Taipei, 11529 Taiwan; 3 Institute of Biomedical Sciences, Academia Sinica, Taipei, 11529 Taiwan; 4 Genomics Research Center, Academia Sinica, Taipei, 11529 Taiwan

**Keywords:** Severe acute respiratory syndrome coronavirus 2 (SARS-CoV-2), Receptor-binding domain (RBD), Neutralizing antibody, Cocktail therapy

## Abstract

**Background:**

Coronavirus disease 2019 (COVID-19) is caused by severe acute respiratory syndrome coronavirus 2 (SARS-CoV-2), an RNA virus with a high mutation rate. Importantly, several currently circulating SARS-CoV-2 variants are associated with loss of efficacy for both vaccines and neutralizing antibodies.

**Methods:**

We analyzed the binding activity of six highly potent antibodies to the spike proteins of SARS-CoV-2 variants, assessed their neutralizing abilities with pseudovirus and authentic SARS-CoV-2 variants and evaluate efficacy of antibody cocktail in Delta SARS-CoV-2-infected hamster models as prophylactic and post-infection treatments.

**Results:**

The tested RBD-chAbs, except RBD-chAb-25, maintained binding ability to spike proteins from SARS-CoV-2 variants. However, only RBD-chAb-45 and -51 retained neutralizing activities; RBD-chAb-1, -15, -25 and -28 exhibited diminished neutralization for all SARS-CoV-2 variants. Notably, several cocktails of our antibodies showed low IC_50_ values (3.35–27.06 ng/ml) against the SARS-CoV-2 variant pseudoviruses including United Kingdom variant B.1.1.7 (Alpha), South Africa variant B.1.351 (Beta), Brazil variant P1 (Gamma), California variant B.1.429 (Epsilon), New York variant B.1.526 (Iota), and India variants, B.1.617.1 (Kappa) and B.1.617.2 (Delta). RBD-chAb-45, and -51 showed PRNT_50_ values 4.93–37.54 ng/ml when used as single treatments or in combination with RBD-chAb-15 or -28, according to plaque assays with authentic Alpha, Gamma and Delta SARS-CoV-2 variants. Furthermore, the antibody cocktail of RBD-chAb-15 and -45 exhibited potent prophylactic and therapeutic effects in Delta SARS-CoV-2 variant-infected hamsters.

**Conclusions:**

The cocktail of RBD-chAbs exhibited potent neutralizing activities against SARS-CoV-2 variants. These antibody cocktails are highly promising candidate tools for controlling new SARS-CoV-2 variants, including Delta.

## Background

Severe acute respiratory syndrome coronavirus 2 (SARS-CoV-2) is responsible for the coronavirus disease 2019 (COVID-19) pandemic, with more than 255 million confirmed cases and  5.14 million deaths as of November 2021. In response to the pandemic, major efforts have been devoted to identifying neutralizing antibodies (Abs) from COVID-19 convalescent patient sera [1–8]. In parallel, mouse immunization and phage display have been utilized to identify other potential therapeutic Abs against SARS-CoV-2 [9–12]. In order to optimize the efficacy of antibody treatments, it may be desirable to develop cocktails of neutralizing Abs that can simultaneously bind different sites of the spike (S) protein receptor binding domain (RBD) and synergistically neutralize SARS-CoV-2 [8, 13]. The emergency use authorizations (EUAs) and rapid deployment of antibody-based prevention and therapeutic agents for COVID-19—such as neutralizing Abs, COVID-19 convalescent sera, messenger RNA vaccines, inactivated vaccines, and viral-vector vaccines—have greatly improved clinical outcomes and helped to prevent progression of infected individuals to intensive care and mortality.

During the evolution of SARS-CoV-2 in humans, variants containing the D614G substitution in the S protein have become dominant due to their increased infectivity and high transmission [14, 15]. Compared to the original SARS-CoV-2 S protein, the D614G mutants are more stable and have a reduced tendency for premature conformation change [16, 17]. However, it widely is accepted that the D614G mutation itself does not increase the severity of disease. More recently, further genetic variants of SARS-CoV-2 have emerged and begun circulating around the world. Among the variants, four that were first identified in the United Kingdom (Alpha, B.1.1.7), South Africa (Beta, B.1.351), Brazil (Gamma, P1) and India (Delta, B.1.617.2) are classified as variants of concern (VOCs). These VOCs have been shown to exhibit increased infectivity, cause more severe disease, reduce the neutralization ability of antibodies generated by previous infection or vaccination, and impair the effectiveness of current therapeutic monoclonal antibodies or vaccines [18–20]. Thus, the VOCs are clinically associated with enhanced transmissibility, increased disease severity, higher risk of death and decreased therapeutic and vaccine effectiveness [21–24]. In particular, Alpha (B.1.1.7), Beta (B.1.351) and Gamma (P1) contain an N501Y mutation in the RBD, which has been shown to increase ACE2 receptor affinity and virulence in mice [25–27]. In addition to N501Y, Beta (B.1.351) and Gamma (P1) include K417N/T and E484K mutations in the RBD, which may also cause important conformational changes. Notably, the binding and neutralization effects of many SARS-CoV-2-neutralizing Abs can be abolished by the K417N and/or E484K mutations [20, 26, 28]. The fourth VOC, Delta (B.1.617.2), harbors L452R and T478K mutations in the RBD and is even more highly transmissible than the other three [29]. In the past several months, the Delta (B.1.617.2) variant has accounted for more than 90% of new cases worldwide. The L452R mutation enhances S protein stability, viral fusogenicity and infectivity, while the T478 mutation likely increases the affinity to ACE2 and impacts the neutralization abilities of monoclonal antibodies and convalescent serum [30, 31]. It has been shown that Delta (B.1.617.2) can fully or partially escape neutralization by antibodies targeting the RBD or N-terminal domain of SARS-CoV-2 S protein [18, 31]. The L452R and E484K/Q mutations are also present in several variants of interest (VOIs), including Epsilon (B.1.427/429) and Iota (B.1.526), which were identified in the United States, and Kappa (B.1.617.1), which was identified in India. These VOIs are predicted to have different transmission, diagnostic, therapeutic, or immune escape profiles than other strains [19]. As the emergence of variant lineages is a major challenge preventing effective control of the COVID-19 pandemic, next-generation vaccines and therapeutic Abs must target variant epitopes, especially those with a high possibility to alter transmission or infectivity. With regard to the efficacies of antibody therapies or vaccines, it will be crucial to understand the implications of antigenic variation on agents for clinical use.

Studies with authentic virus or pseudovirus suggest that neutralization by some antibodies and immune sera may be diminished for variants expressing mutated S protein [18, 20, 28, 32–37]. In our previous study, we generated six neutralizing chimeric Abs (chAbs) against the RBD of SARS-CoV-2 S protein, all of which exhibited potent neutralizing capabilities in vitro and in vivo. Furthermore, the prophylactic and therapeutic efficacies of these chAbs or antibody cocktails were confirmed in SARS-CoV-2-infected mouse and hamster models [38, 39]. Variants of SARS-CoV-2 that carry mutations in the RBD could affect the binding and neutralizing abilities of our antibodies. Therefore, we sought to evaluate the neutralizing activities of our chAbs with pseudotyped virus of SARS-CoV-2 variants, including Alpha (B.1.1.7), Beta (B.1.351), Gamma (P1), Epsilon (B.1.427/429), Iota (B.1.526) and Kappa (B.1.617.1) and Delta (B.1.617.2). Our data showed that most of the chAbs maintain neutralizing ability against these variants, which correlated with the abilities of the chAbs to bind full S protein. Moreover, a cocktail of therapeutic chAbs targeting separate epitopes on the receptor binding motif (RBM) of SARS-CoV-2 S protein may increase therapeutic efficacy and decrease the potential for emergence of virus escape mutants. The prophylactic and therapeutic potentials of these antibodies and their combinations were confirmed in SARS-CoV-2 hamster infection models, wherein injection of the therapeutic chAb cocktail markedly reduced the virus titers, underscoring their potential for use in prevention and treatment of COVID-19. Thus, cocktails of our chAbs may provide effective tools to tackle the emergence of new variants harboring multiple S protein mutations.

## Materials and methods

### Antibody binding to SARS-CoV-2 S protein variants by ELISA

ELISA plates were coated with 0.5 μg/ml SARS-CoV-2 variant S-His protein or EpEX-His protein (negative control) in 0.1 M NaHCO_3_ (pH 8.6) buffer at 4 °C overnight, followed by blocking with PBS containing 1% bovine serum albumin (BSA) at RT for 2 h. After blocking, the wells were washed twice with PBS; then, the plates were stored at − 20 °C. RBD-chAb or rabbit anti-His Ab was added at a concentration of 30 ng/ml in each well, and the plate was incubated for 1 h at room temperature. The plates were washed with PBS containing 0.1% Tween-20 (PBST_0.1_) three times and then incubated for 1 h with Peroxidase AffiniPure Goat Anti-human IgG (H + L) (Jackson ImmunoResearch) (1:4000 dilution) or Peroxidase AffiniPure Goat Anti-rabbit IgG (H + L) (Jackson ImmunoResearch) (1:10,000 dilution), as appropriate. After three washes with PBST_0.1_, signal was produced using 3,3′5,5'-Tetramethylbenzidine (TMB) color development (TMBW-1000–01, SURMODICS). The reaction was stopped with 3 N HCl, and absorbance was measured at 450 nm by ELISA reader (Versa Max Tunable Microplate Reader; Molecular Devices).

### Pseudovirus neutralization assay

The pseudovirus neutralization assays were performed using HEK293T cells that stably expressed human ACE2 (HEK293T/hACE2); SARS-CoV-2 pseudotyped lentivirus expressing full-length S protein was provided by the National RNAi Core Facility (Academia Sinica, Taiwan). HEK293T/hACE2 cells were seeded into 96-well white plate (Corning Costar) at a density of 1 × 10^4^ cells per well, and cultivated for 16 h at 37 °C. Serial dilutions of RBD-chAbs were pre-incubated with 1000 TU SARS-CoV-2 pseudovirus in a 96-well microtiter plate for 1 h at 37 °C, and then, the mixtures were added to pre-seeded HEK293T/hACE2 cells for 24 h at 37 °C. The pseudovirus-containing culture medium was removed and replaced with 50 μL/well DMEM for an additional 48-h incubation. Next, 50 μL ONE-Glo luciferase reagent (Promega) was added to each well for 3-min incubation at 37 °C. The luminescence was measured with a microplate spectrophotometer (Molecular Devices). Inhibitions of 0% or 100% were respectively calculated based on pseudovirus only and cells only. The half maximal inhibitory concentration (IC_50_) was calculated by nonlinear regression using Prism software version 8.1.0 (GraphPad Software Inc.). The average IC_50_ value for each antibody was determined from at least two independent experiments.

### Plaque reduction neutralization test (PRNT)

RBD-chAbs were serially diluted in PBS and pre-incubated with 100 plaque-forming units (PFU) SARS-CoV-2 for 1 h at 37 °C. The mixtures were added to pre-seeded Vero E6 cells for 1 h at 37 °C. The viral-containing culture medium was removed and replaced with DMEM containing 2% FBS and 1% methyl-cellulose for an additional 4-day incubation. The cells were fixed with 10% formaldehyde overnight and stained with 0.5% crystal violet for 20 min. The plates were then washed with tap water, and plaque numbers formed at each dilution were counted. Virus without RBD-chAb served as a control. Each experiment was performed in triplicate. Plaque reduction was calculated as: Inhibition percentage = 100 × [1 – (plaque number incubated with chAb/plaque number without chAb)]. The 50% plaque reduction (PRNT_50_) value was calculated with Prism software. The SARS-CoV-2 variants used in this study, i.e., Alpha (hCoV-19/Taiwan/792/2020), Gamma (hCoV-19/Taiwan/906/2021) and Delta (hCoV-19/Taiwan/1144/2021), were obtained from Taiwan Centers for Disease Control (CDC). The PRNT assay was performed at the BSL-3 facility in the Institute of Biomedical Sciences, Academia Sinica.

### In vivo prophylactic and therapeutic assays for SARS-CoV-2 infection

Hamster models of SARS-CoV-2 infection were used to evaluate the potency of neutralizing chAbs against SARS-CoV-2 RBD in vivo. Each hamster was first intraperitoneally administered RBD-chAb antibody or normal huamn IgG as a control. Twenty-four hours later, each hamster was intranasally inoculated with 10^4^ PFU SARS-CoV-2 (strain: TCDC#4). At day 3 after virus challenge, the hamsters were sacrificed to harvest lung tissues to quantify the viral load. Lung tissues were weighed and homogenized for two cycles of 2 min in the SpeedMill PLUS equipment (Analytik Jena AG) or RLT buffer (RNeasy mini kit, Qiagen). After tissue homogenization, the supernatant was collected for the TCID_50_ assay or RNA extraction. Homogenates were serial tenfold dilutions and applied to a Vero-E6 cell monolayer in 1% FBS/DMEM for 4 days. The plates were observed for cytopathic effects and used to calculate TCID_50_, the amount of virus causing cytopathic effects in 50% of inoculated cells.

The therapeutic activities of chAbs cocktails in hamsters were evaluated after intranasal inoculation with 10^4^ PFU virus. Mixtures of RBD-chAb-15 and -45 were intraperitoneally injected into hamsters at day 2 after SARS-CoV-2 inoculation. The hamsters were sacrificed to collect lung tissue at day 3 post-challenge. All animal studies were carried out in accordance with the established guidelines for the ethical use and care of animals provided by the Institutional Animal Care and Use Committee (IACUC) at Academia Sinica, Taiwan. All experiments involving animals were approved by the IACUC (protocol 20–05-147). The SARS-CoV-2 strains used in this study are clinical isolates of the WT strain (hCoV-19/Taiwan/4/2020) and Delta variant (hCoV-19/Taiwan/1144/2021) and were obtained from the Taiwan CDC.

### Real-time RT-PCR for SARS-CoV-2 RNA quantification

SARS-CoV-2 viral burden in the lung tissues was measured by Taqman quantitative real-time RT-PCR with primers designed to target the envelope (E) gene of SARS-CoV-2 genome, as previously described [40]. Forward primer E-Sarbeco-F1 (5’-ACAGGTACGTTAATAGTTAATAGCGT-3’) and reverse primer E-Sarbeco-R2 (5’-ATATTGCAGCAGTACGCACACA-3’), in addition to the probe E-Sarbeco-P1 (5’-FAM-ACACTAGCCATCCTTACTGCGCTTCG-BBQ-3’) were used. RNA was extracted from lung homogenate supernatants using RNeasy Mini Kit (QIAGEN, Germany) according to the manufacturer's instructions. The RNA sample (5 μL) was added in a total 25 μL mixture using Superscript III one-step RT-PCR system with Platinum Taq Polymerase (Thermo Fisher Scientific, USA). The final reaction mix contained 400 nM forward and reverse primers, 200 nM probe, 1.6 mM of deoxy-ribonucleoside triphosphate (dNTP), 4 mM magnesium sulphate, 50 nM ROX reference dye and 1 μL of enzyme mixture from the kit. The cycling conditions were performed with a one-step PCR protocol: 55 °C for 10 min for cDNA synthesis, followed by 3 min at 94 °C and 45 amplification cycles at 94 °C for 15 s and 58 °C for 30 s. Data were collected and calculated with the Applied Biosystems 7500 Real-Time PCR System (Thermo Fisher Scientific, USA). To establish a qPCR standard curve, a synthetic 113-bp oligonucleotide fragment was used to estimate copy numbers of viral genome.

## Results

### Binding and neutralizing abilities of anti-RBD chAbs

**Fig. 1:**
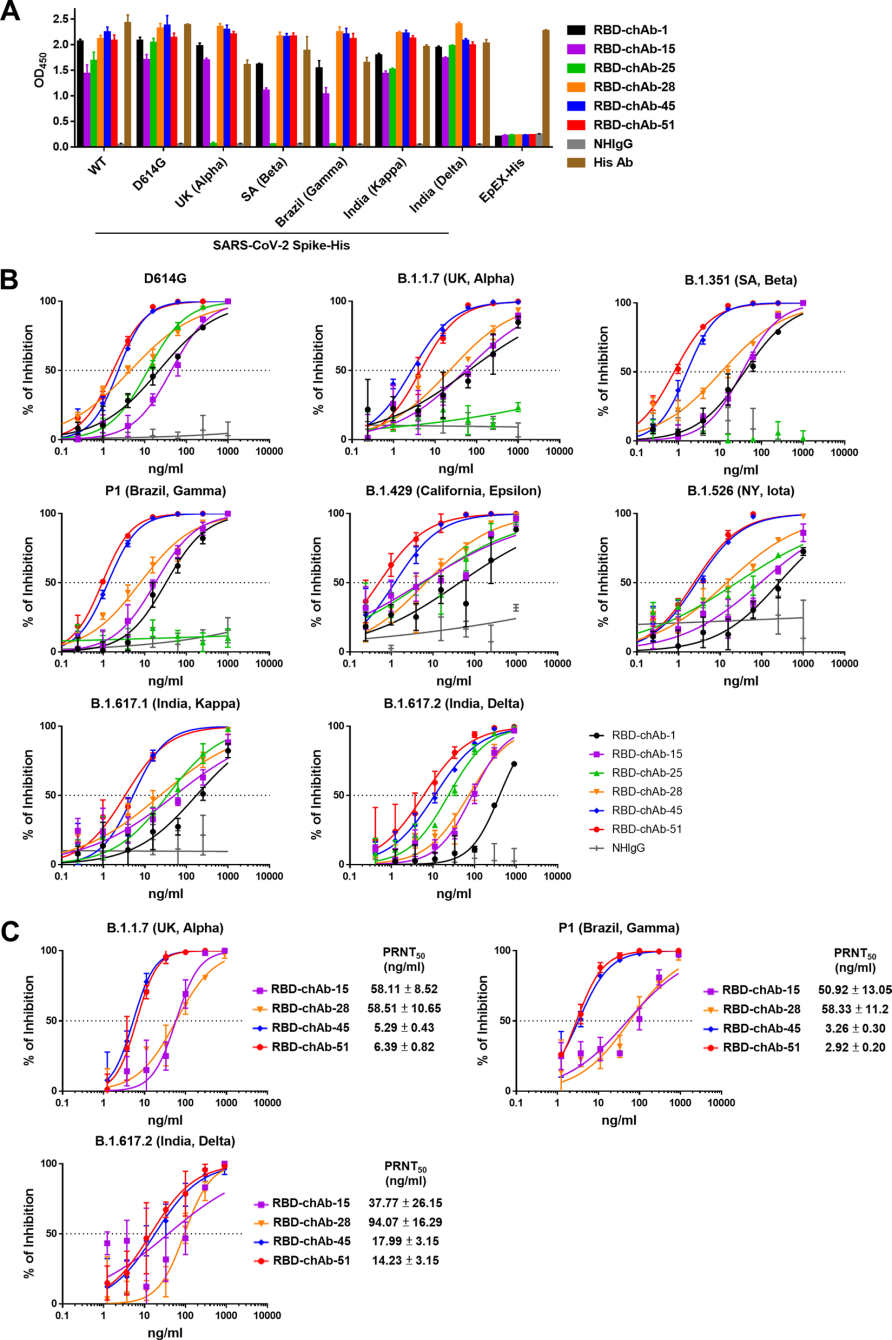
Single chAbs binding and neutralizing capacities toward SARS-CoV-2 variants. **A** The binding of anti-RBD chAbs to S-His protein of SARS-CoV-2 variants was probed by ELISA. Data for each RBD-chAb are representative of three independent experiments. Each assay was performed in triplicate, and data points represent the mean. **B** Neutralization assay of SARS-CoV-2 variant pseudoviruses with RBD-chAbs. Data for each RBD-chAb are representative of three independent neutralization experiments. Each assay was performed in triplicate, and data points represent the mean. **C** Neutralizing RBD-chAbs inhibit SARS-CoV-2 variants, Alpha, Gamma and Delta; infection was assessed by PRNT. The PRNT_50_ value was calculated with Prism software. Each assay was performed in triplicate and all data points are shown, along with the mean ± SD

VOCs contain common mutations, such as K417N/T, L452R, T478K, E484K, in their RBD domains; therefore, we first used ELISA to examine the binding ability of our RBD-chAbs to recombinant S protein of SARS-CoV-2 variants. The results showed that most of the RBD-chAbs maintained binding ability to S protein from SARS-CoV-2 variants; the only exception was RBD-chAb-25 (Fig. 1A). In line with the antibody recognition sites identified in our previous study, only the binding of RBD-chAb-25 was significantly diminished when tested against the S proteins of Alpha, Beta, and Gamma variants containing the N501Y mutation. This result suggested that besides RBD-chAb-25, most of our neutralizing Abs might retain activity against these VOCs. Our previous work showed that RBD-chAb-25 and -45 could simultaneously bind to the RBD of SARS-CoV-2 S protein using cryo-EM, and the combination exhibited a synergistic effect compared to single chAbs when used as a prophylactic treatment [39]. Therefore, we further examined the neutralizing abilities of our six most potent RBD-chAbs toward several SARS-CoV-2 variant pseudoviruses. Pseudovirus neutralization assays revealed that RBD-chAb-25 exhibited poor neutralizing abilities for the United Kingdom variant B.1.1.7 (Alpha), South African variant B.1.351 (Beta) and Brazil variant P1 (Gamma), all of which contain the N501Y mutation (Fig. 1B). However, the rest of the RBD-chAbs retained their abilities to neutralize several common variants, including the United Kingdom variant B.1.1.7 (Alpha), South African variant B.1.351 (Beta), Brazil variant P1 (Gamma), California variant B.1.429 (Epsilon), New York variant B.1.526 (Iota) and India variants B.1.617.1 (Kappa) and B.1.617.2 (Delta) (Fig. 1B). RBD-chAb-45 and -51 exhibited lower IC_50_ values and better neutralizing activities than the other four RBD-chAbs for all variants (Table 1). Additionally, we evaluated the neutralization potentials of the RBD-chAbs by conducting the in vitro plaque reduction neutralization test (PRNT). RBD-chAb-45 and -51 could effectively block infection with authentic SARS-CoV-2 Alpha, Gamma and Delta variants, with PRNT_50_ values of less than 18 ng/ml; RBD-chAb-15 and -28 were worse at neutralizing the authentic SARS-CoV-2 Alpha and Gamma variants, with PRNT_50_ values ranging from 50 to 94 ng/ml (Fig. 1C).

**Table 1:** Half-maximal inhibitory concentrations (IC_50_) values for single RBD-chAbs against pseudoviruses of SARS-CoV-2 variants

	SARS-CoV-2 pseudovirus IC_50_ (ng/ml)			
	D614G	B.1.1.7 (UK, Alpha)	B.1.351 (SA, Beta)	P1 (Brazil, Gamma)
RBD-chAb-1	24.35 ± 5.55	33.46 ± 17.9	46.71 ± 10.98	17.09 ± 7.72
RBD-chAb-15	21.25 ± 11.13	39.73 ± 16.66	30.13 ± 12.03	7.18 ± 5.46
RBD-chAb-25	9.92 ± 2.04	Non-N	Non-N	Non-N
RBD-chAb-28	9.55 ± 4.16	11.29 ± 5.93	16.52 ± 6.03	4.18 ± 1.80
RBD-chAb-45	1.58 ± 0.54	2.06 ± 0.58	2.68 ± 0.84	0.76 ± 0.14
RBD-chAb-51	1.30 ± 0.27	3.07 ± 0.72	1.26 ± 0.58	0.70 ± 0.19

	SARS-CoV-2 pseudovirus IC_50_ (ng/ml)			
	B.1.429 (California, Epsilon)	B.1.526 (NY, Iota)	B.1.617.1 (India, Kappa)	B.1.617.2 (India, Delta)
RBD-chAb-1	21.6 ± 11.13	428.2 ± 166.3	117.7 ± 55.64	429.3 ± 36.55
RBD-chAb-15	11.72 ± 5.40	87.89 ± 5.09	67.71 ± 15.84	103.6 ± 16.57
RBD-chAb-25	6.51 ± 0.73	91.95 ± 66.85	42.27 ± 7.27	35.5 ± 12.14
RBD-chAb-28	3.28 ± 1.71	44.07 ± 29.58	60.62 ± 37.4	94.13 ± 22.07
RBD-chAb-45	0.91 ± 0.16	4.23 ± 1.51	5.95 ± 0.72	15.51 ± 4.58
RBD-chAb-51	1.32 ± 0.75	2.28 ± 0.08	4.55 ± 1.2	8.04 ± 2.11

Data are from three independent experiments and are shown as mean ± SEM

*Non-N* non-neutralizing

### Neutralizing abilities of anti-RBD chAbs in combination

**Fig. 2:**
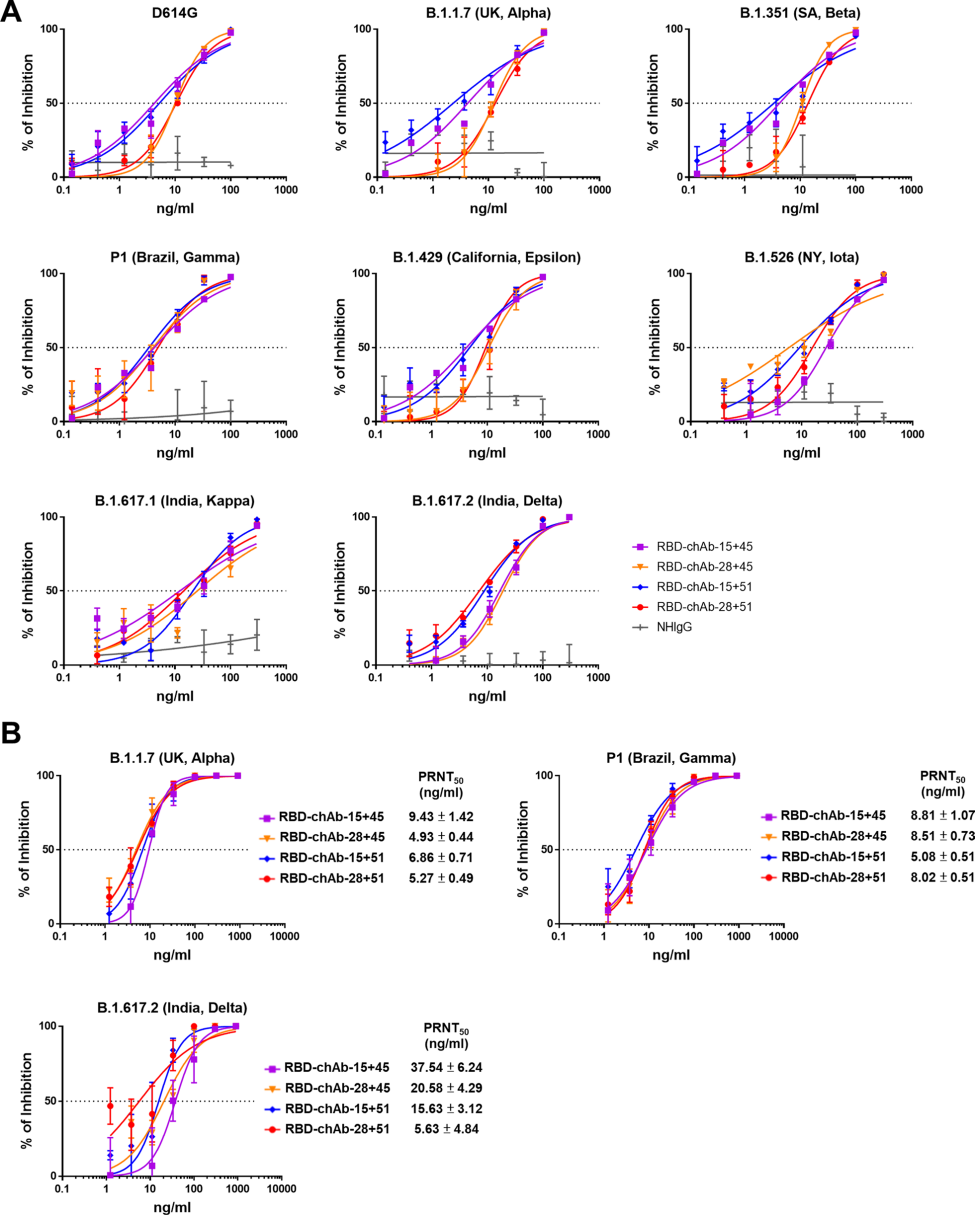
Neutralization of SARS-CoV-2 variants by RBD-chAb combinations. **A** Neutralization assays testing RBD-chAb-15 or -28 combined with -45 or -51 against SARS-CoV-2 variant pseudoviruses. Each assay was performed in triplicate; data points represent the mean. Data for each RBD-chAb are representative of at least two independent neutralization experiments. **B** Neutralizing RBD-chAb-15 or -28 combined with -45 or -51 inhibits SARS-CoV-2 variants, Alpha, Gamma and Delta; infection was assessed by PRNT. The PRNT_50_ value was calculated with Prism software. Each assay was performed in triplicate, and all data points are shown, along with the mean ± SD

Previously, we found that RBD-chAb-45 and -51 share overlapping epitopes according to an ELISA-based competition-binding assay [39]. In addition, RBD-chAb-15 and -28 have highly similar epitopes, and RBD-chAb-25 has an epitope that partially overlaps with those of RBD-chAb-15 and -28. However, only RBD-chAb-25 loses its neutralizing ability against SARS-CoV-2 variant pseudoviruses with the N501Y mutation [39]. To evaluate the neutralizing abilities of cocktails containing RBD-chAbs with different epitopes, we performed neutralization tests using SARS-CoV-2 variant pseudoviruses. Combinations of RBD-chAb-15 or -28 with RBD-chAb-45 or -51 exhibited high neutralizing activities toward different SARS-CoV-2 pseudoviruses, including Alpha, Beta, Gamma, Epsilon, Iota, Kappa and Delta variants (Fig. 2A). The RBD-chAb cocktails showed low IC_50_ values ranging from 3 to 27 ng/ml (Table 2). To evaluate the RBD-chAbs cocktail neutralization potential against the authentic SARS-CoV-2 Alpha, Gamma and Delta variants, we performed the PRNT and showed that RBD-chAb-15 or -28 combined with RBD-chAb-45 or -51 displayed the high potencies against the authentic virus; the PRNT_50_ values were less than 38 ng/ml (Fig. 2B).

**Table 2:** Half-maximal inhibitory concentrations (IC_50_) values for RBD-chAb combinations against pseudoviruses of SARS-CoV-2 variants

	SARS-CoV-2 pseudovirus IC_50_ (ng/ml)			
	D614G	B.1.1.7 (UK, Alpha)	B.1.351 (SA, Beta)	P1 (Brazil, Gamma)
RBD-chAb-15 + 45	7.41 ± 2.22	7.28 ± 1.40	4.27 ± 0.95	5.27 ± 2.82
RBD-chAb-28 + 45	10.69 ± 1.83	11.77 ± 0.80	8.25 ± 1.43	5.40 ± 0.72
RBD-chAb-15 + 51	4.76 ± 0.48	4.66 ± 1.25	3.55 ± 0.78	3.35 ± 0.55
RBD-chAb-28 + 51	10.06 ± 2.71	12.72 ± 2.81	8.73 ± 2.84	4.19 ± 0.92

	SARS-CoV-2 pseudo virus IC_50_ (ng/ml)			
	B.1.429 (California, Epsilon)	B.1.526 (NY, Iota)	B.1.617.1 (India, Kappa)	B.1.617.2 (India, Delta)
RBD-chAb-15 + 45	6.31 ± 2.33	21.7 ± 5.36	19.35 ± 7.08	25.69 ± 8.73
RBD-chAb-28 + 45	11.04 ± 0.90	6.81 ± 0.49	27.06 ± 1.81	21.55 ± 2.32
RBD-chAb-15 + 51	5.86 ± 0.87	8.34 ± 0.2	20.29 ± 0.86	10.13 ± 1.25
RBD-chAb-28 + 51	12.15 ± 2.67	11.44 ± 2.85	18.61 ± 4.24	14.22 ± 6.98

Data are from at least two independent experiments and are shown as mean ± SEM

### Prophylactic effect of RBD-chAb in SARS-CoV-2-infected hamsters

**Fig. 3:**
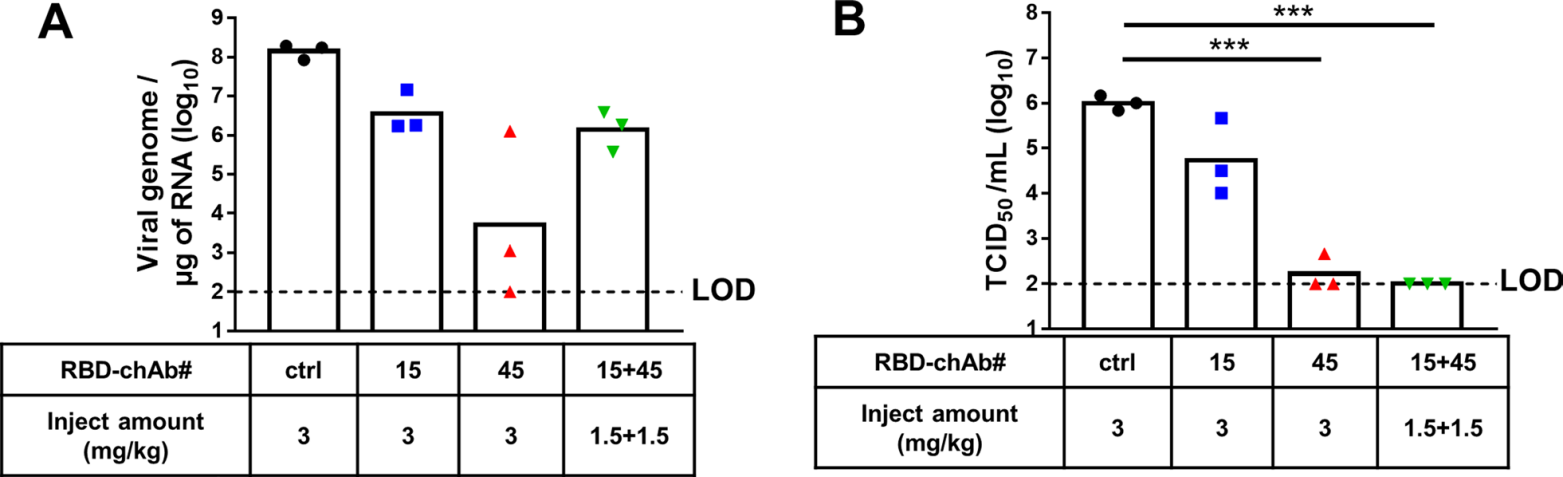
Prophylactic effects of neutralizing RBD-chAbs against SARS-CoV-2 infection. One day prior to intranasal (i.n.) challenge of WT SARS-CoV-2, each group of hamsters was given a single intraperitoneal injection of 3 mg/kg RBD-chAb-15 (n = 3), 3 mg/kg RBD-chAb-45 (n = 3), total of 3 mg/kg RBD-chAb-15 combined with RBD-chAb-45 (n = 3), or 3 mg/kg NHIgG isotype control (n = 3). On day 3 after virus inoculation, lung samples were collected for analysis. **A** The viral load in the lungs of treated hamsters was determined by qRT-PCR. **B** The viral load in the lungs of treated hamsters was determined by median tissue culture infectious dose per ml (TCID_50_/ml). Statistical differences were determined by two-tailed Student *t* test. ***P < 0.001

Next, we studied our antibodies in a hamster model of mild human SARS-CoV-2 infection that has been utilized in the development of therapies [41]. We examined the efficacies of two low-dose RBD-chAbs (RBD-chAb-15 and -45) individually and as a cocktail in the hamsters (Fig. 3). Single intraperitoneal injections of one RBD-chAb alone (3 mg/kg) or RBD-chAb-15 and -45 in combination (1.5 mg/kg each) were made one day prior to WT SARS-CoV-2 infection and conferred dramatic protection; infectious SARS-CoV-2 titers were determined from the lung tissue at the third day post-infection. The viral genome RNA (as measured by RT-qPCR) could still be detected at the end of the experiment (Fig. 3A). Nevertheless, the infectious SARS-CoV-2 titers were just above the limit of detection (LOD, 1 × 10^2^ TCID_50_/ml) for almost all hamsters in the RBD-chAb cocktail-treated group (1.5 mg/kg of each RBD-chAb-15 and -45) at 3 days post-infection (Fig. 3B).

### Therapeutic effect of RBD-chAb cocktail in SARS-CoV-2-infected hamsters

**Fig. 4:**
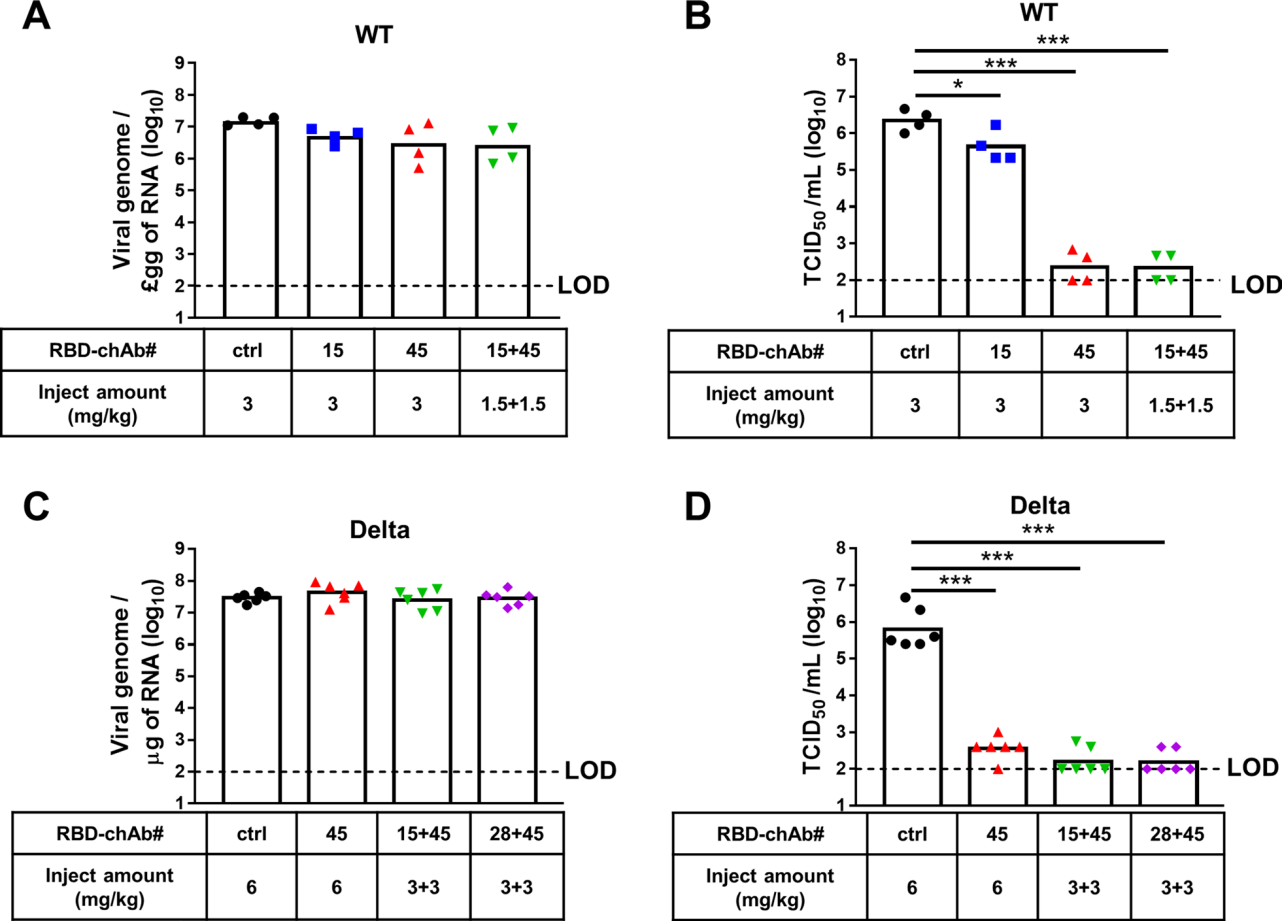
Therapeutic effects of neutralizing RBD-chAbs against SARS-CoV-2 infection. **A**, **B**, One day after to intranasal (i.n.) challenge of WT SARS-CoV-2, each group of hamsters was given a single intraperitoneal injection of 3 mg/kg RBD-chAb-15 (n = 4), 3 mg/kg RBD-chAb-45 (n = 4), a total of 3 mg/kg RBD-chAb-15 combined with RBD-chAb-45 (n = 4), or 3 mg/kg NHIgG isotype control (n = 4). **C**, **D**, One day after to intranasal (i.n.) challenge with Delta SARS-CoV-2 variant, each group of hamsters was given a single intraperitoneal injection. Injections contained: 6 mg/kg RBD-chAb-45 (n = 6), a total of 6 mg/kg RBD-chAb-15 combined with RBD-chAb-45 (n = 6), a total of 6 mg/kg RBD-chAb-28 combined with RBD-chAb-45 (n = 6), or 6 mg/kg NHIgG isotype control (n = 6). **A** and **C**, On day 3 after virus inoculation, the viral load in the lungs of treated hamsters was determined by qRT-PCR. **B**, **D** On day 3 after virus inoculation, the viral load in the lungs of treated hamsters was determined by median tissue culture infectious dose per ml (TCID_50_/ml). Statistical differences were determined by two-tailed Student *t* test. *P < 0.05, ***P < 0.001

We next tested the therapeutic effects of the antibody cocktail administered after SARS-CoV-2 infection in the hamster model (Fig. 4). We treated hamsters with RBD-chAb-15 or -28 combined with RBD-chAb-45 at one day post-inoculation with WT (Fig. 4A, B) or Delta (Fig. 4C, D) SARS-CoV-2 (intranasal). The infectious SARS-CoV-2 titers were then determined from lung tissue at the third day post-infection. The levels of viral genome RNA were measured by RT-qPCR at the end of the experiment (Fig. 4A, C). Similar to the results of the antibody prophylaxis experiments, the infectious SARS-CoV-2 titers were close to the limit of detection (LOD, 1 × 10^2^ TCID_50_/ml) for all hamsters receiving RBD-chAb cocktail treatments (1.5 mg/kg each of RBD-chAb-15 and -45 for WT SARS-CoV-2; 3 mg/kg each of either RBD-chAb-15 or -28 combined with RBD-chAb-45 for Delta SARS-CoV-2) at 3 days post-infection (Fig. 4B, D). Collectively, these data demonstrated remarkable prophylactic and therapeutic effects of combined RBD-chAb-15 and -45 in SARS-CoV-2-infected hamsters.

## Discussion

SARS-CoV-2 is an RNA virus with a high mutation rate, which results in the rapid emergence of variants. Identified variants with high transmissibility or that cause increased rates of severe disease or death are classified as VOCs and include: B.1.1.7 (Alpha), B.1.351 (Beta), P.1 (Gamma) and B.1.617.2 (Delta) [42]. A major public health concern is that new SARS-CoV-2 variants may be resistant to neutralizing antibodies induced by infection or vaccination, as well as therapeutic antibodies developed against original SARS-CoV-2. Here, we report that our previously identified antibodies, RBD-chAb-45 and -51, retain high binding ability for all tested SARS-CoV-2 variant pseudoviruses, including four VOCs (Fig. 1). Because the epitope for RBD-chAb-25 includes N501 in the S protein, the antibody had reduced binding ability toward variants with the N501Y mutation [including B.1.1.7 (Alpha), B.1.351 (Beta) and P1 (Gamma)] (Fig. 1). However, RBD-chAb-25 still retained the ability to recognize other variants (Fig. 1). Combinations of RBD-chAbs showed neutralization ability for all tested SARS-CoV-2 variants in the pseudovirus neutralization assay (Fig. 2). Therefore, our six RBD-chAbs can be used strategically to create cocktail therapies against various SARS-CoV-2 mutant strains. The prophylactic and therapeutic potentials of a cocktail including RBD-chAb-15 and -45 were verified in SARS-CoV-2-infected hamster animal models (Figs. 3, 4).

Up to now, hundreds of mutations have been identified in the S protein of SARS-CoV-2. Some of these mutations might confer resistance to vaccines and neutralizing Abs due to local or global changes in protein conformation [20, 42]. For example, bamlanivimab (LY-CoV555), a human IgG1 targeting the RBD of S protein, was discovered by Eli-Lilly and AbCellera from single antigen-specific B cells of a COVID-19 convalescent patient [43]. Bamlanivimab received an EUA from the U.S. FDA to treat mild to moderate COVID-19 in adults and pediatric patients on November 9, 2020 [44], and it exhibits high neutralization potency against the B.1.1.7 (Alpha) variant strain. However, bamlanivimab is unable to block B.1.351 (Beta), P.1 (Gamma), B.1.429 (Epsilon), B.1.526 (Iota) and B.1.617.1 (Kappa) variants, due to the presence of E484K/Q or L452R mutations [20, 27, 28, 45]. Because many of the common circulating SARS-CoV-2 viral variants are resistant to the drug, the U.S. FDA revoked the EUA for use of bamlanivimab alone to treat COVID-19 on April 9, 2021.

Etesevimab (LY-CoV016) is a human IgG targeting the RBD of S protein that was identified from single B cells from a COVID-19 convalescent patient [7]. The combination of bamlanivimab and etesevimab received an EUA from the U.S. FDA, as it can neutralize B.1.1.7 (Alpha); however, B.1.351 (Beta) and P.1 (Gamma) variants with the K417N/T mutation are resistant to the cocktail of etesevimab and bamlanivimab [20, 28]. Notably, the B.1.351 (Beta) and P.1 (Gamma) variants are also resistant to casirivimab [20, 28]. In contrast, all of our potent neutralizing RBD-chAbs except RBD-chAb-25 could effectively block B.1.351 (Beta) and P.1 (Gamma) variants in the pseudovirus neutralization assay.

The SARS-CoV-2 B.1.617.2 variant, also known as Delta, was first identified in October 2020 in India and became the dominant strain around the world by July 2021 [18]. The B.1.617.2 (Delta) variant is up to 60% more transmissible than the B.1.1.7 (Alpha) variant, with an R_0_ estimated at 5–7 [29]. Planas et al. reported that sera from people who had received one dose of Pfizer or AstraZeneca vaccines barely inhibited variant B.1.617.2 (Delta). Furthermore, the levels of neutralizing antibodies in people with two vaccine doses were 3–5-fold lower when tested against B.1.617.2 (Delta) compared to B.1.1.7 (Alpha) [18]. Additionally, bamlanivimab does not have appreciable antiviral activity against B.1.617.2 (Delta) due to the L452R mutation, but etesevimab retains neutralization ability against the variant [18, 45]. Our potent neutralizing antibodies, RBD-chAb-1, -15, -25 and -28, also exhibited partially reduced neutralizing ability against the B.1.617.2 (Delta) variant. However, according to the pseudovirus neutralization assay, RBD-chAb-45 and -51 retained high neutralizing capabilities toward the B.1.617.2 (Delta) variant, with IC_50_ values of about 8–15 ng/ml for single RBD-chAb treatments and 10–25 ng/ml for combination treatments.

According to cryo-EM structures of the UK (Alpha) variant S protein in combination with RBD-chAb-15 and -45, the two antibodies have non-overlapping epitopes and can simultaneously bind to the same upward pointing RBD. Further, three each of the RBD-chAb-15 and -45 molecules can bind to the three RBDs in a SARS-CoV-2 UK variant S protein trimer [38]. This cryo-EM structure of RBD-chAbs and S protein suggested that RBD-chAb-15 and -45 could be useful as a cocktail therapy for COVID-19, and we demonstrated that the cocktail of RBD-chAbs exhibited good neutralizing capability with low IC_50_ values in SARS-CoV-2 variant pseudovirus neutralizing experiments. Furthermore, the antibody cocktail of RBD-chAb-15 and -45 exhibited prophylactic and therapeutic effects in SARS-CoV-2-infected hamsters. Therefore, we predict that RBD-chAb-15 and -45 may be used strategically to create cocktail therapies against multiple SARS-CoV-2 variants.

## Conclusions

COVID-19 is caused by SARS-CoV-2, and several currently circulating SARS-CoV-2 variants are associated with loss of efficacy for both vaccines and neutralizing antibodies. Here, we analyzed the binding of six highly chAbs to the spike proteins of SARS-CoV-2 variants with ELISA, and assessed their neutralizing abilities with pseudovirus and authentic SARS-CoV-2 variants. Notably, several cocktails of our antibodies showed low IC_50_ and PRNT_50_ values against the pseudovirus and authentic SARS-CoV-2 variants, respectively. Furthermore, the antibody cocktail of RBD-chAb-15 and -45 exhibited potent prophylactic and therapeutic effects in WT and Delta SARS-CoV-2 variant-infected hamsters. Thus, these antibody cocktails are highly promising candidate tools for controlling new SARS-CoV-2 variants.

## Data Availability

All materials are available by the corresponding author.

## Abbreviations

COVID-19: Coronavirus disease 2019
SARS-CoV-2: Severe acute respiratory syndrome coronavirus 2
S: Spike
RBD: Receptor-binding domain
RBM: Receptor binding motif
VOC: Variant of concern
VOI: Variant of interest
chAbs: Chimeric antibodies
PRNT: Plaque reduction neutralization test
IC_50_: Half maximal inhibitory concentration
CDC: Centers for disease control
PFU: Plaque-forming units
LOD: Limit of detection
EUA: Emergency use authorization
